# Pharmacokinetics and safety of highly variable valsartan in single-pill combination with amlodipine versus its generic formulation: a randomized, three-cycle, three-sequence, partially replicated crossover phase I bioequivalence clinical trial

**DOI:** 10.3389/fphar.2023.1264321

**Published:** 2023-09-07

**Authors:** Bo Qiu, Haojing Song, Congyang Ding, Xue Sun, Runxuan Du, Haotian Yang, Wanjun Bai, Zhanjun Dong

**Affiliations:** Phase I Clinical Trial Laboratory, Hebei General Hospital, Shijiazhuang, China

**Keywords:** bioequivalence, hypertension, valsartan/amlodipine, safety, pharmacokinetics

## Abstract

Valsartan/amlodipine (I) is a single-pill combination (SPC) of an angiotensin II receptor blocker (ARB) and a calcium channel blocker (CCB) for treating hypertension. A clinical trial was performed to demonstrate that the test and reference valsartan/amlodipine formulations were bioequivalent under fasting and postprandial conditions. Participants were randomly divided into three sequences at a ratio of 1:1:1 for three-cycle, reference formulation replicated, crossover administration. The average bioequivalence (ABE) and reference-scaled average bioequivalence (RSABE) methods were used to evaluate BE using the main pharmacokinetic (PK) parameters. Overall, 45 eligible participants were enrolled in the postprandial trial, which was consistent with the fasting trial. For valsartan, the RSABE method was used to evaluate the BE of C_max_, while the ABE method was applied to evaluate the BE of AUC_0–t_ and AUC_0–∞_. Both point estimates and 95% upper confidence bound met the BE criteria. For amlodipine, the ABE method was performed, and the 90% confidence intervals of the geometric mean ratios (GMR) for C_max_ and AUC_0–72 h_ were all within 80%–125%, with the BE criteria being met. Therefore, the two formulations are bioequivalent and have similar safety profiles in healthy Chinese subjects.

**Clinical trial registration:** [http://www.chinadrugtrials.org.cn/index.html], identifier [CTR20210214].

## 1 Introduction

Hypertension is a common and growing public health issue, with a worldwide prevalence of 40.8% and a control rate of 32.3% (Khan et al., 2020). Without effective control, hypertension is a major risk factor for several diseases, including cardiovascular disease (CVD), cerebrovascular disease, and chronic kidney disease. The burden of CVD has increased over the past several decades and is a major concern in China (Lu et al., 2017). Thus, any improvement in blood pressure control is expected to result in considerable savings in healthcare expenditure (Lim et al., 2012; Kizilirmak et al., 2015). According to the Chinese Cardiovascular Health and Disease Report, there are 245 million people with hypertension in China. The prevalence of hypertension is increasing, and the situation remains grim in China. Most patients with hypertension require at least two anti-hypertensive agents for blood pressure control. An initial combined treatment for essential hypertension has favorable effects and is safe, which is one of the basic principles of anti-hypertensive treatment (Franklin et al., 2009; Tsioufis et al., 2020). However, polypharmacy may significantly decrease patient compliance, leading to a worse condition and increasing mortality rates (Benford et al., 2012).

The International Society of Hypertension’s Global Practice Guidelines for Hypertension (2020) recommend combination therapy for all patients with hypertension, except for those with low-risk grade 1 hypertension or frailty. Combination therapy, particularly SPC, is the first-line anti-hypertensive treatment option (Unger et al., 2020). Current guidelines recommend combining two anti-hypertensive drugs with different mechanisms preferably as an SPC because treatment simplification promotes patient adherence (Mancia et al., 2013). Moreover, adherence to cardioprotective treatments can reduce morbidity and mortality (Simpson et al., 2006; Corrao et al., 2011).

There are various combinations for SPC include the following: ARB + CCB, ARB + thiazide diuretic, angiotensin-converting enzyme inhibitors (ACE-I) + CCB, ACE-I + thiazide diuretic, CCB + thiazide diuretic and CCB + beta-blocker (Parati et al., 2021). ARB combined with CCB have complementary anti-hypertensive mechanisms, allowing adverse reactions to be mitigated Blood pressure reduction through combined ARB and CCB in patients with hypertension has significant benefits in terms of reducing major CV events and mortality compared with the other combinations (Kizilirmak et al., 2015).

Amlodipine, a dihydropyridine CCB used to treat hypertension, reaches its peak concentration between 6 and 8 h after oral dosing. Amlodipine is mostly absorbed, metabolized in the liver, and excreted in the urine (Khodadoustan et al., 2017). Valsartan, an ARB with selectivity for the type 1 receptor subtype, is predominantly eliminated via biliary excretion (Waldmeier et al., 1997). Maximal plasma concentrations occur 2–4 h after oral administration, and only approximately 20% of valsartan is metabolized (Wu et al., 2020). Within-subject variability in the C_max_ and area under the curve for valsartan was reportedly significant (Zakeri-Milani et al., 2011). Considering these findings, a three-sequence, three-cycle, crossover, partially replicative design was applied in this study to determine within-subject variability for the reference (R) formulation. The within-subject standard deviation (S_WR_) of the R formulation was calculated before the bioequivalence assessment, and the standard ABE was applied if S_WR_ <0.294 for any primary PK parameter (C_max_, AUC_0–t_, AUC_0–∞_). The RSABE method was applied to the BE evaluation if S_WR_ ≥0.294 (CV_WR_% ≥30%) for any primary PK parameter, according to the National Medical Products Administration (NMPA) Guidelines for Human Bioavailability and Bioequivalence Studies for Pharmaceuticals (Wang et al., 2020).

The effectiveness and safety of Valsartan/amlodipine (I) for patients with hypertension have been well established through clinical trials (Zhang et al., 2017). However, the high cost of original products imposes a financial burden on patients. Generic products have lower costs than original products providing a potential method to overcome patients’ economic burden and the accessibility of anti-hypertensive agents will be improved. Bioequivalence studies comparing generic to original products are required for marketing a new generic product by the NMPA. A generic drug is considered to be bioequivalent to the reference drug if the rate and extent of absorption of the two products do not show any signifcant difference. In addition, the pharmacokinetics and safety of Valsartan/amlodipine (I) in Chinese can be explored through the BE study.

Therefore, the current study aimed to investigate the PK properties and BE of two formulations of valsartan/amlodipine tablet (I) (80/5 mg) in healthy participants under fasting and postprandial conditions to gain a better understanding of their *in vivo* characteristics, and to provide evidence for the test (T) formulation to be marketed in China.

## 2 Methods

### 2.1 Study drug

Valsartan/amlodipine tablet (I) (80/5 mg per tablet, batch number: XA20072001), which was produced and provided by Shijiazhuang No.4 Pharmaceutical Co. Ltd, and valsartan/amlodipine tablet (I) (80/5 mg per tablet, batch number: BNH61), which is marketed under the brand name EXFORGE^®^ and produced by Novartis Pharma Schweiz, were used as the T and R formulations, respectively, for the assessment of PK and BE studies.

### 2.2 Ethics approval and study population

The study was conducted at the Phase I Clinical Research Center of Hebei General Hospital between March and June 2021 and registered at the Drug Clinical Trial Registration and Information Disclosure Platform (Registration No. CTR20210214). The study was performed in accordance with the Good Clinical Practice requirements of the NMPA, Declaration of Helsinki, and International Conference on Harmonization Good Clinical Practice Guidelines. The protocol and amendments were approved by the Ethics Committee of Hebei General Hospital (approval No. 2020-11, 2020-11-01).

Healthy men and women aged ≥18 years with a body mass index between 19.0 and 26.0 kg/m^2^ (≥45 kg for females and ≥50 kg for males) were eligible for the study. They underwent a comprehensive medical examination, including routine physical examination, measurement of vital signs, medical history, laboratory tests, 12-lead electrocardiography, and chest radiography to assess their health status and reveal clinically relevant diseases. All examination items mentioned above should have no clinically significant abnormalities. Patients with the following conditions were not eligible for participation in this study: acute or chronic disease not applicable to this study, alcohol or smoking abuse, drug abuse in the past year, allergy-prone constitution, and allergy to any ingredient in the valsartan/amlodipine tablet (I). Participants who had taken any medication within 28 days prior to the first dose, participated in any clinical trial within the past 3 months, suffered blood loss, or donated blood of ≥200 mL were excluded. Participants who were pregnant, breastfeeding, or were likely to become pregnant were also excluded. Additionally, considering the drug mechanism and safety, participants with postural hypotension were excluded.

Written informed consent was obtained from each participant before the study was conducted and included information on the objectives, procedures, and risk-benefit analysis. A withdrawal option was available to participants at any time during the study period.

### 2.3 Study design

This was a single-center, randomized, open-label, three-cycle, three-sequence, partially replicated crossover bioequivalence study, and each cycle was followed by a 14-day washout period. The study consisted of two independent trials: a fasting trial and a postprandial trial. SAS statistical software (v9.4) was used to generate a random number table that assigned participants of the fasting trial into sequences A (TRR), B (RTR), or C (RRT) in a 1:1:1 ratio. The participants in the postprandial trial were randomly assigned into sequences D (TRR), E (RTR), or F (RRT) in the same manner.

Each participant was administered a single dose of the T or R formulation under light-proof conditions, and 240 mL of warm water was orally administered thereafter. In the fasting trial, participants fasted overnight for at least 10 h, while in the postprandial trial, they consumed a standard high-calorie and high-fat (800–1,000 kcal: protein, 150 kcal; carbohydrates, 250 kcal; and fat, 500–600 kcal) breakfast 30 min before drug administration. Water intake was restricted to 1 h before and 2 h after drug administration. The upper body was kept upright for 4 h after drug administration. Throughout the study period, alcohol consumption, strenuous activities, and smoking were prohibited. A standardized meal was provided at 4 and 10 h after treatment.

### 2.4 PK analysis

Blood samples were collected into coded, pre-cooled K_2_-EDTA anticoagulation tubes pre-dose (0 h, baseline) and 0.5, 1, 1.5, 2, 2.5, 3, 3.5, 4, 4.5, 5, 5.5, 6, 7, 8, 9, 10, 11, 12, 24, 48, and 72 h post-dose followed by centrifugation at 2,600 g at 4°C for 10 min within 1 h of collection. Plasma was collected and stored at −70°C ± 10°C within 2 h of collection until their use for analysis. All blood samples were light-proofed throughout collection, processing, and storage.

The plasma concentrations of valsartan and amlodipine were determined by the Triple Quad 5500 liquid chromatography-tandem mass spectrometry system. Gradient elution and multiple reaction monitoring were used to quantify target compounds. The analytes were chromatographed using an ExionLC AD and an ACQUITY UPLC BEH C18 column (2.1 mm × 50 mm; 1.7 µm). The mobile phase consisted of mobile phases A (0.2% formic acid in water) and B (0.2% formic acid in acetonitrile) at a flow rate of 0.5 mL/min. An MS/MS Triple Quad 5500 was used to analyze the mass. Data acquisition and analysis were performed using Analyst 1.6.3.

The PK analysis set comprised all participants randomly assigned to any group who completed periods 1–3 without any major protocol deviations. The primary PK endpoints for amlodipine were the peak concentration (C_max_) and the area under the curve from time zero to the last measurable concentration (AUC_0–72 h_). For valsartan, the peak concentration (C_max_), area under the curve from time zero to the last measurable concentration (AUC_0–t_) and AUC from time zero to observed infinity (AUC_0–∞_) were the primary PK endpoints. The terminal half-life of the analyte in plasma (t_1/2_), time of maximum plasma concentration (T_max_), and terminal rate constant (λ_z_) were the secondary endpoints.

### 2.5 BE analysis

Owing to the expected high intra-individual variation for valsartan, a partially replicated design was used in the current study based on the within-subject variability of the R formulation, and the RSABE method recommended by the Food and Drug Administration (FDA) and NMPA was applied. The within-subject standard deviation (S_WR_) of the R formulation was calculated before the bioequivalence assessment, and the standard ABE was applied if S_WR_ <0.294 for any primary PK parameter (C_max_, AUC_0–t_, AUC_0–∞_), which corresponds to a within-subject variability (CV_WR_%) of <30%. If the 90% confidence interval (CI) for the test/reference GMR of the above PK parameter was within 80.00%–125.00%, then BE was confirmed.

The RSABE method was applied to the BE evaluation if S_WR_ ≥0.294 (CV_WR_% ≥30%) for any primary PK parameter. In this case, the 95% upper confidence bound for ${\left({\bar{Y}}_{T}-{\bar{Y}}_{R}\right)}^{2}$ *—θs* ^*2*^_*WR*_ was calculated based on Howe’s Approximation I, where ${\bar{Y}}_{T}$ and ${\bar{Y}}_{R}$ are the natural log-transformed AUC or C_max_ mean values for the test and reference formulations, respectively. *θ =* $\left[\frac{\ln1.25}{\sigma w0}\right]$ ^*2*^ represent the threshold of the corrected bioequivalence and σw0 = 0.25 is the regulatory constant set by the US FDA and China NMPA. For any given primary PK parameter (s), bioequivalence was concluded if the 95% upper confidence bound (Critbound) for ${\left({\bar{Y}}_{T}-{\bar{Y}}_{R}\right)}^{2}$ *— θs* ^*2*^_*WR*_ is ≤ 0 and the test/reference GMR (point estimate) falls within 80.00%–125.00%.

The ABE criteria were used to evaluate the BE of amlodipine, which was determined if the differences between the compared parameters were insignificant (*p* > 0.05) and if the 90% CI for the GMR of C_max_ and AUC_0–72 h_ fell within 80%−125%, respectively. Furthermore, in accordance with the NMPA regulatory guidelines, logarithm-transformed PK parameters were analyzed using multivariate analysis of variance to assess the effects of sequence, formulation, and period.

### 2.6 Safety analysis

Various laboratory tests, including biochemistry, hematology, and urinalysis were performed, and adverse events (AEs) and vital signs were monitored, to assess the safety of both formulations. Vital signs, including axillary temperature, blood pressure (systolic and diastolic), and pulse rate, were recorded 1 h before administration and at 2, 5, 8, 12, 24, and 48 h post-dose at each treatment visit. Laboratory and physical examinations along with an electrocardiogram were performed at baseline and 72 h after the second administration. AEs were coded according to the preferred term and system organ class in the Medical Dictionary for Regulatory Activities. The severity of adverse events was graded according to the National Cancer Institute Common Terminology Criteria for Adverse Events (NCI CTCAE 5.0), version 5.0. In addition to medical observations, the participants spontaneously reported any adverse events that occurred during the study.

### 2.7 Statistical analysis

Phoenix WinNonlin Software v8.0, was used to calculate the PK parameters using the non-compartmental method, and individual plasma concentration–time curves were constructed. The main PK parameters derived from the plasma concentration–time curve were subjected, after logarithmic transformation, to multivariate analysis of variance that included participants nested within the sequence as a random effect and sequence, formulation, and period as fixed effects. Two-sided t-tests were performed. Statistical analysis of the T_max_ was performed using the non-parametric Wilcoxon two-sample test. Descriptive statistics were used for the PK parameters, and count or grade data were expressed as frequencies and percentages. Statistical analyses were performed using the statistical software package SAS, V9.4. *p* < 0.05 was considered statistically significant.

## 3 Results

### 3.1 Subject demographics

**FIGURE 1:**
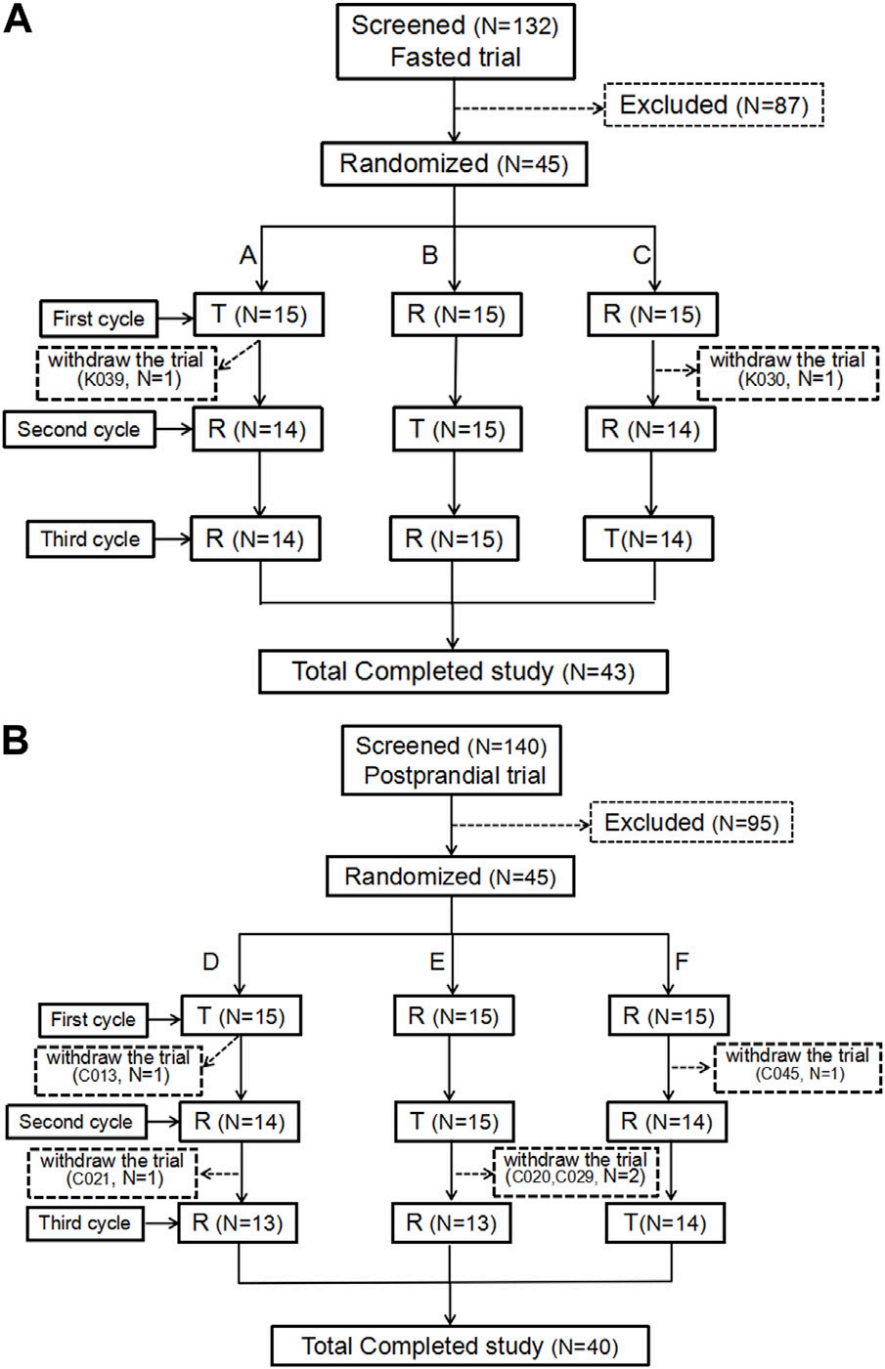
Patient flow chart. Flow chart of the subjects in the fasting state **(A)**. Flow chart of the subjects in the postprandial state **(B)**. N, the number of subjects.

During the trial screening for fasting and postprandial conditions, there were 132 and 140 participants, respectively. The fasting and postprandial trials each enrolled 45 participants (17 women/28 men and 16 women/29 men, respectively). Two participants in the fasting group and five participants in the postprandial group withdrew from the trial. Figure 1 show the screening and inclusion distributions of the participants in the two groups.

In the fasting group, participant K039 withdrew from the trial due to AEs (headache, palpitations, and sweating) 5.5 h after the first cycle and after 12 blood samples were collected. Participant K030 was withdrawn from the trial because the urine drug screening was positive for morphine before the second cycle of administration. Additionally, two participants were enrolled in the full analysis set (FAS), safety analysis set (SS), pharmacokinetic parameter analysis set (PKPS), and pharmacokinetic concentration analysis set (PKCS). Meanwhile, participant K039 was not included in the bioequivalence analysis set (because of an incomplete first cycle).

In the postprandial group, 45 participants were assigned to the FAS, PKPS, PKCS, and SS sets. Participant C013 withdrew from the trial for personal reasons after blood collection within 24 h of the first cycle. Participants C020, C021, and C029 completed the first two cycles and decided to withdraw from the trial prior to the third cycle. Participant C045 only completed the first cycle and withdrew from the trial for personal reasons prior to the second cycle. No adverse events in the postprandial group led to withdrawal from the study.

The baseline characteristics of each participant are presented in Table 1. All participants met the inclusion criteria.

**TABLE 1:** Demographic baseline.

Variable	Fasting trial	Postprandial trial
Age(years)		
Mean ± SD	33.29 (8.03)	33.04 (8.63)
Median(Q1; Q3)	34 (26,40)	33 (24,38)
Min; Max	18, 47	19, 50
Sex n (%)		
Male	28 (62.22)	29 (64.44)
Female	17 (37.78)	16 (35.56)
Ethnicity n (%)		
ethnic Han	45 (100.00)	45 (100.00)
Others	0 (0.00)	0 (0.00)
Height (cm)		
Mean ± SD	165.74 (7.83)	167.07 (8.43)
Median(Q1; Q3)	168.0 (159.0,171.0)	167.5 (161.5,171.0)
Min; Max	152.5, 184.0	148.5, 189.5
Weight (kg)		
Mean(Std)	62.97 (7.40)	63.55 (8.45)
Median(Q1; Q3)	61.2 (57.3,69.0)	62.7 (57.4,69.5)
Min; Max	50.0, 77.8	49.8, 78.6
BMI (kg/m2)		
Mean(Std)	22.89 (1.89)	22.70 (1.90)
Median(Q1; Q3)	23.2 (21.0,24.6)	22.6 (21.0,24.5)
Min; Max	19.4, 25.9	19.2, 25.8

### 3.2 PK results

**FIGURE 2:**
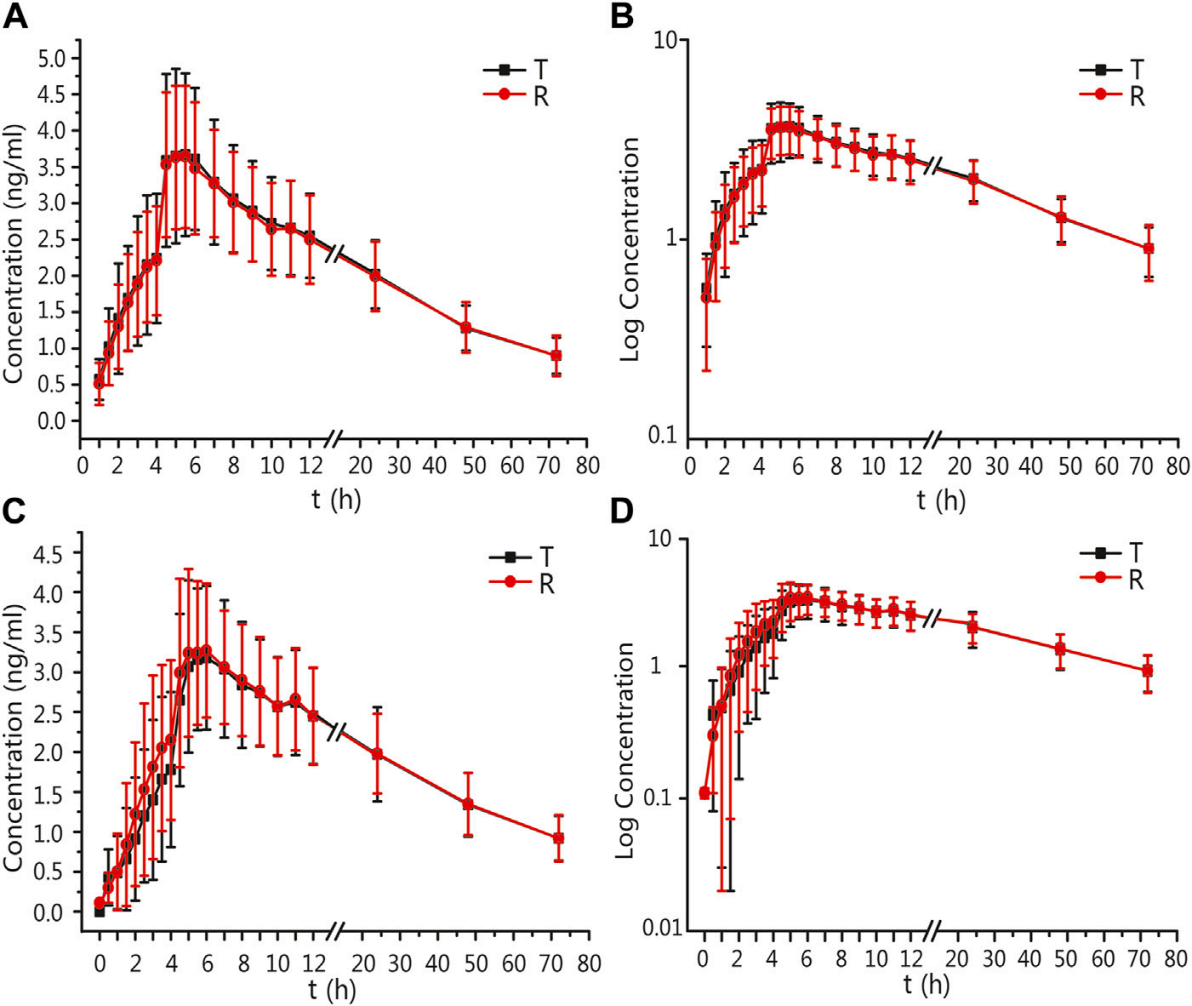
PK analysis of amlodipine of T and R formulations. Mean blood concentration (±SD) time curve after oral T and R formulations during fasting: arithmetic mean **(A)** and log transformation **(B)**. Mean blood concentration (±SD) time curve after oral T and R formulations postprandially: arithmetic mean **(C)** and log transformation **(D)**.

**FIGURE 3:**
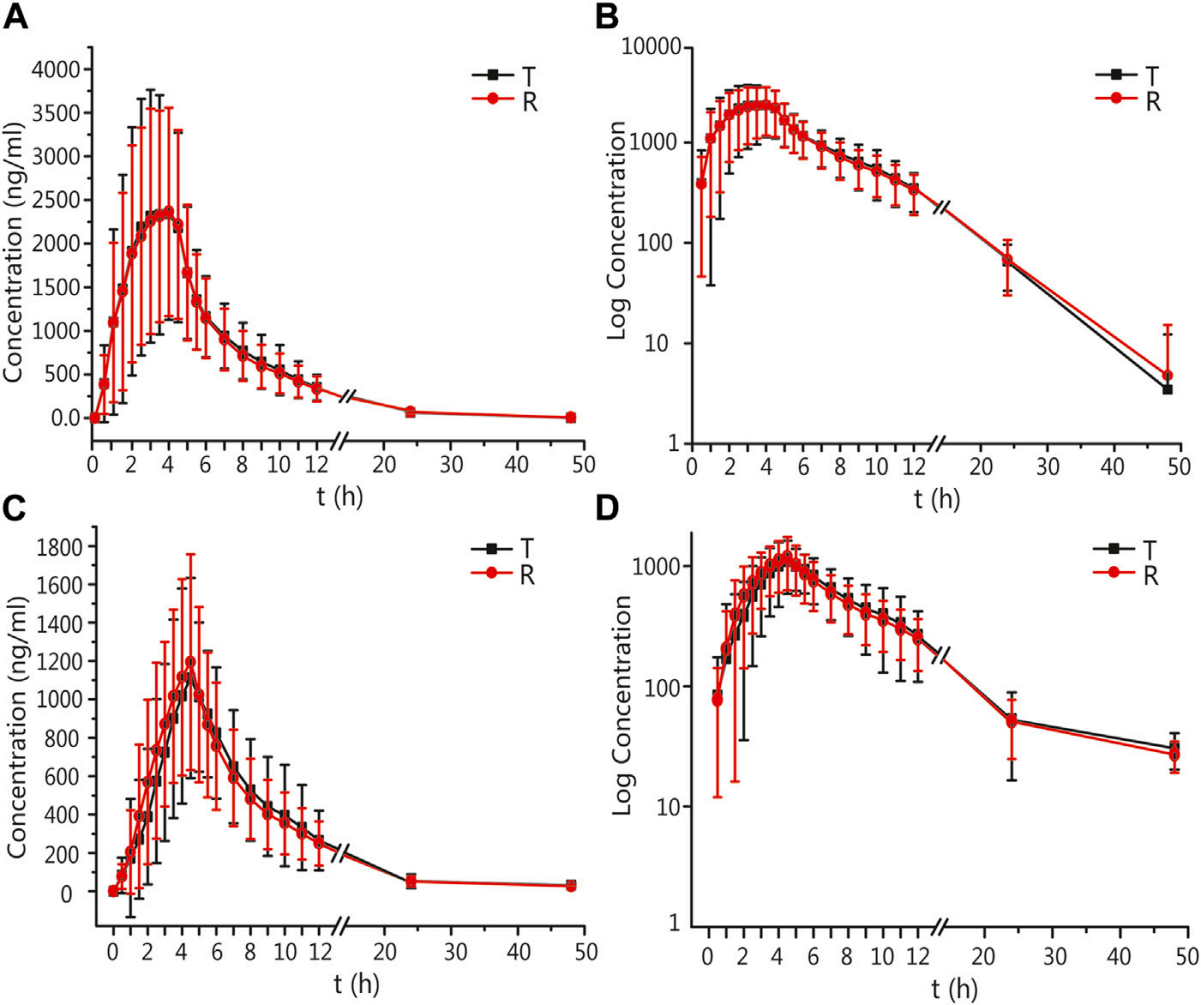
PK analysis of valsartan of T and R formulations. Mean blood concentration (±SD) time curve after oral T and R formulations during fasting: arithmetic mean **(A)** and log transformation **(B)**. Mean blood concentration (±SD) time curve after oral T and R formulations postprandially: arithmetic mean **(C)** and log transformation **(D)**.

The mean ± standard deviation (SD) plasma drug concentration–time curves for amlodipine and valsartan under fasting and postprandial conditions after completing the three cycles are shown in Figure 2 and Figure 3.

In the fasting group, the main PK parameters for amlodipine of the T and R formulations were as follows: The C_max_ and AUC_0–72 h_ values were 4.03 ± 1.02 ng/mL and 3.94 ± 0.92 ng/mL, and 121.48 ± 26.80 h*ng/mL and 120.44 ± 27.58 h*ng/mL, respectively. The main PK parameters for valsartan of the T and R formulations were as follows: The C_max_, AUC_0–t_, and AUC_0–∞_ values were 2818.82 ± 1419.96 ng/mL and 2799.64 ± 1385.07 ng/mL, 16035.91 ± 6912.77 h*ng/mL and 15873.30 ± 6751.62 h*ng/mL, and 16496.43 ± 6959.21 h*ng/mL and 16309.66 ± 6889.28 h*ng/mL, respectively.

In the postprandial group, the main PK parameters of amlodipine of the T and R formulations were as follows: The C_max_ and AUC_0–72 h_ values were 3.58 ± 0.80 ng/mL and 3.67 ± 0.86 ng/mL, and 117.42 ± 29.13 h*ng/mL and 119.65 ± 28.57 h*ng/mL, respectively. The main PK parameters for valsartan of the T and R formulations were as follows: The C_max_, AUC_0–t_, and AUC_0–∞_ values were 1376.98 ± 473.07 ng/mL and 1364.56 ± 534.53 ng/mL, 8409.56 ± 3315.33 h*ng/mL and 8329.16 ± 2911.29 h*ng/mL, and 8910.50 ± 3225.82 h*ng/mL and 8717.39 ± 3012.41 h*ng/mL, respectively. The detailed PK parameters are shown in Tables 2, 3.

**TABLE 2:** The PK parameters of amlodipine after oral T and R formulations under fasting and postprandial conditions.

PK parameters	Fasting trial		Postprandial trial	
	Mean ± SD		Mean ± SD	
	T	R	T	R
T_max_(h)	4.99(4.49, 12.03) (N = 44)	4.99(4.49, 12.01) (N = 87)	5.49(3.99, 24.00) (N = 44)	5.49(1.99, 10.99) (N = 84)
C_max_(ng/mL)	4.03 ± 1.02 (N = 44)	3.94 ± 0.92 (N = 87)	3.58 ± 0.80 (N = 44)	3.67 ± 0.86 (N = 84)
AUC_0–72h_*(h·ng/mL)	121.48 ± 26.80 (N = 43)	120.44 ± 27.58 (N = 87)	117.42 ± 29.13 (N = 43)	119.65 ± 28.57 (N = 84)
λz(h^-1^)	0.02 ± 0.00 (N = 43)	0.02 ± 0.00 (N = 87)	0.02 ± 0.00 (N = 43)	0.02 ± 0.00 (N = 84)
t_1/2_(h)	41.77 ± 11.12 (N = 43)	41.77 ± 11.08 (N = 87)	44.88 ± 9.74 (N = 43)	44.26 ± 11.30 (N = 84)

**TABLE 3:** The PK parameters of valsartan after oral T and R formulations under fasting and postprandial conditions.

PK parameters	Fasting trial		Postprandial trial	
	Mean ± SD		Mean ± SD	
	T	R	T	R
T_max_(h)	3.24(1.49, 8.99) (N = 44)	2.99(0.99, 6.99) (N = 87)	4.49(0.99, 9.99) (N = 44)	4.49(0.99, 5.99) (N = 84)
C_max_(ng/mL)	2818.82 ± 1419.96 (N = 44)	2799.64 ± 1385.07 (N = 87)	1376.98 ± 473.07 (N = 44)	1364.56 ± 534.53 (N = 84)
AUC_0-t_(h·ng/mL)	16035.91 ± 6912.77 (N = 44)	15873.30 ± 6751.62 (N = 87)	8409.56 ± 3315.33 (N = 44)	8329.16 ± 2911.29 (N = 84)
AUC_0-∞_(h·ng/mL)	16496.43 ± 6959.21 (N = 44)	16309.66 ± 6889.28 (N = 87)	8910.50 ± 3225.82 (N = 43)	8717.39 ± 3012.41 (N = 84)
λz(h^-1^)	0.15 ± 0.05 (N = 44)	0.13 ± 0.03 (N = 87)	0.14 ± 0.04 (N = 44)	0.14 ± 0.03 (N = 84)
t_1/2_(h)	5.06 ± 1.36 (N = 44)	5.58 ± 1.78 (N = 87)	5.62 ± 1.80 (N = 43)	5.46 ± 1.96 (N = 84)

The period, sequence, and formulation factors may be affect the equivalence of the test preparation and reference preparation in bioequivalence trials. To assess whether these external factors have an impact on the current study, we used a linear Mixed Model to perform a multivariate analysis of variance after log transformation of each main parameter in the fasting or postprandial state, respectively. The results demonstrated that the mixed effects model included period, sequence, and formulation factors that did not interfere with the conclusions of the study either in the fasting or postprandial state. (Table 4) (*p* > 0.05 both in the fasting and postprandial).

**TABLE 4:** The results of variance analysis of the main PK parameters after logarithmic transformation.

Effect factor	Postprandial trial (*P*)					Fasting trial (*P*)				
	amlodipine		valsartan			amlodipine		valsartan		
	LnC_max_	LnAUC_0–72h_	LnC_max_	LnAUC_0-t_	LnAUC_0-∞_	LnC_max_	LnAUC_0–72h_	LnC_max_	LnAUC_0-t_	LnAUC_0-∞_
Sequence	0.5097	0.0692	0.7369	0.9508	0.9463	0.6572	0.6790	0.8559	0.6989	0.6826
Formulation	0.2704	0.1295	0.8284	0.9397	0.8841	0.3032	0.8367	0.9751	0.8369	0.8922
Period	0.4570	0.6437	0.7112	0.6453	0.6545	0.0850	0.6795	0.1102	0.5317	0.5642

### 3.3 BE results

**FIGURE 4:**
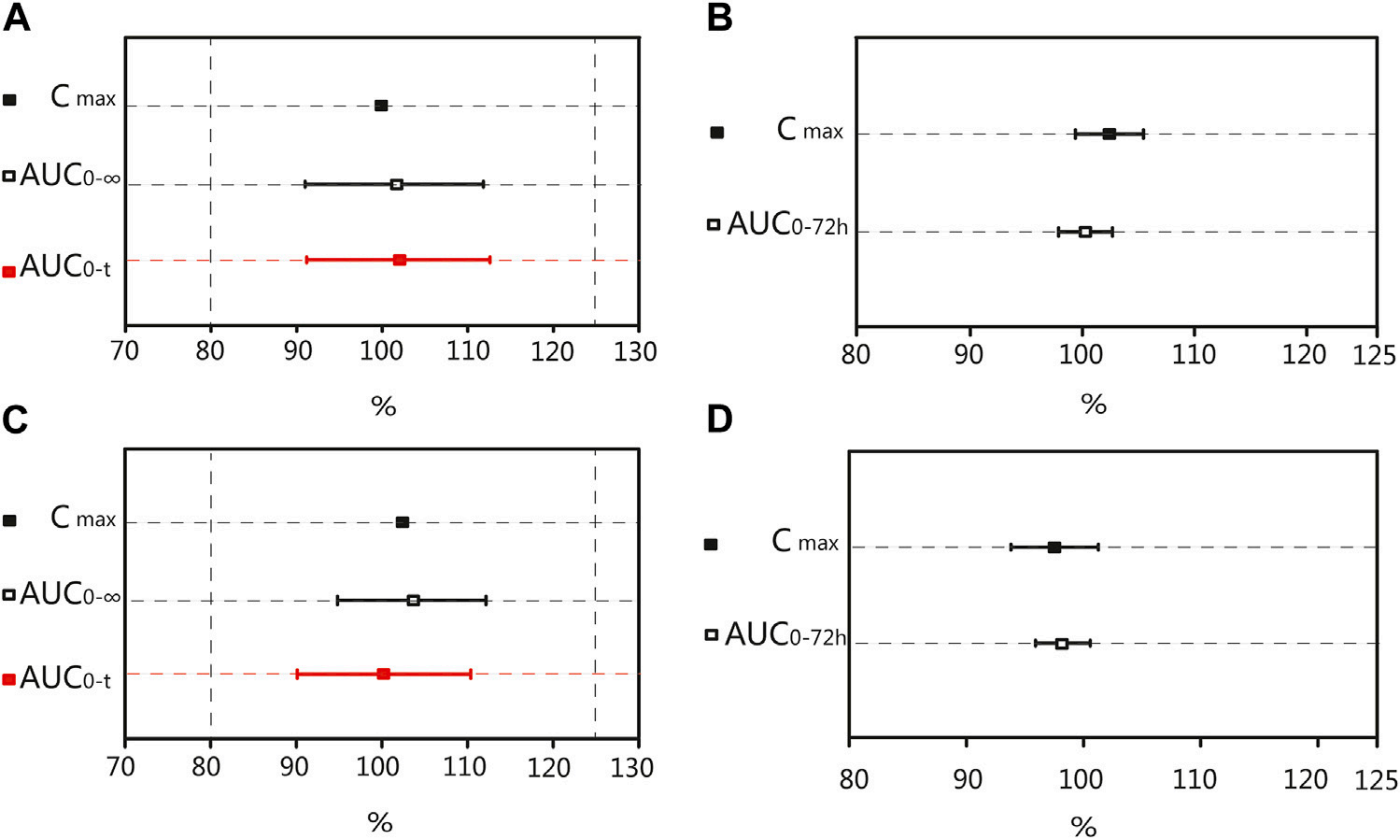
Bioequivalence analysis of T and R formulations. The bioequivalence analysis of valsartan of T and R formulations during fasting **(A)** shows the ratio range of AUC_0-t_ and AUC_0–∞_ of T and R formulations with 90% CIs and the Point Estimate of C_max_. The bioequivalence analysis of amlodipine of T and R formulations during fasting **(B)** shows the ratio range of the main PK parameters of amlodipine of T and R formulations with 90% CIs. The bioequivalence analysis of valsartan of T and R formulations during postprandial **(C)** shows the ratio range of AUC_0-t_ and AUC_0–∞_ of T and R formulations with 90% CIs and the Point Estimate of C_max_. The bioequivalence analysis of amlodipine of T and R formulations during postprandial **(D)** shows the ratio range of the main PK parameters of amlodipine of T and R formulations with 90% CIs. (bioequivalence was declared if the Point Estimate and 90% CIs were within the prespecified acceptable ranges of 80%–125%). C_max_: the maximum observed drug concentration in the plasma; AUC_0–∞_: AUC of the analyte in the plasma over the time interval from time zero to infinity; AUC_0-t_: AUC of the analyte in the plasma over the time interval from time zero to the last measurable concentration.

After oral administration of the R formulation in the fasting trial, the CV_WR_ of C_max_, AUC_0–t_, and AUC_0–∞_ for valsartan were 34.68%, 29.03%, and 28.04%, respectively. The CV_WR_ of C_max_ was greater than 30%; therefore, the RSABE criterion was used for equivalence evaluation. The point estimate value of the C_max_ for valsartan was 99.45%, which fell within 80.00%–125.00%, while the Critbound of the C_max_ for valsartan was −0.0628 < 0, meeting the BE criteria.The CV_WR_ of AUC_0–t_ and AUC_0–∞_ were less than 30%; thus, the ABE criterion was adopted to evaluate the bioequivalence for these PK parameters. The GMR of the AUC_0–t_ for valsartan was 101.32%, and the 90% CI was 91.17%–112.59%. The GMR of the AUC_0–∞_ for valsartan was 100.88%, and the 90% CI was 91.00%–111.82% (Table 5). The AUC_0–t_ and AUC_0–∞_ of valsartan were within the BE limits of 80.00%–125.00% (Figure 4A). BE analysis of amlodipine was performed using the C_max_ and AUC_0–72 h_. The GMR of C_max_ for amlodipine was 102.37%, and the 90% CI was 99.38%–105.45%. The GMR of the AUC_0–72 h_ for amlodipine was 100.27%, and the 90% CI was 97.89%–102.69% (Table 5). The AUC_0–72 h_ and C_max_ of amlodipine were within the BE limits of 80.00%–125.00% (Figure 4B).

**TABLE 5:** Results of the equivalence determination of the T and R formulations in the fasting trial.

Bioequivalence determination of amlodipine										

PK parameters	T		R		GMR (%)	CV_WR_ (%)	90%CI		Power (%)	Bioequivalence criteria met?
	N	GM	N	GM						
C_max_(ng/mL)	43	3.91	87	3.81	102.37	15.1	99.38∼105.45		>99.99	YES
AUC_0–72h_(h·ng/mL)	43	116.39	87	116.08	100.27	9.61	97.89∼102.69		>99.99	YES
Bioequivalence determination of valsartan (ABE)										

PK parameters	T		R		GMR (%)	CV_WR_ (%)	90%CI		Power (%)	Bioequivalence Criteria met?
	N	GM	N	GM						
AUC_0-t_(h·ng/mL)	43	14596.54	87	14406.31	101.32	29.03	91.17∼112.59		94.13	YES
AUC_0-∞_(h·ng/mL)	43	14963.30	87	14832.55	100.88	28.04	91.00∼111.82		95.78	YES
Bioequivalence determination of valsartan (RSABE)										

PK parameters	T		R		S_WR_	CV_WR_ (%)	Point Estimate	Critbound		Bioequivalence Criteria met?
	N	GM	N	GM						
C_max_(ng/mL)	43	2467.60	87	2470.82	0.3370	34.68	99.45	−0.0628		YES

After oral administration of the R formulation in the postprandial trial, the CV_WR_ of C_max_, AUC_0–t_, and AUC_0-∞_ for valsartan were 30.36%, 21.58% and 20.19%, respectively. The CV_WR_ of C_max_ was greater than 30%; therefore, the RSABE criterion was used for equivalence evaluation. The point estimate value of the C_max_ for valsartan was 102.47%, which fell within 80.00%–125.00%, and the Critbound of the C_max_ for valsartan was −0.0453 < 0, which met the criteria for BE. The CV_WR_ of AUC_0–t_ and AUC_0–∞_ were less than 30%; thus, the ABE criterion was adopted. The GMR of AUC_0–t_ for valsartan was 99.72%, and the 90% CI was 90.10%–110.37%. The GMR of the AUC_0–∞_ for valsartan was 99.43%, and the 90% CI was 90.34%–109.42% (Table 6). The AUC_0–t_ and AUC_0–∞_ of valsartan were within the BE limits of 80.00%–125.00% (Figure 4C). BE analysis of amlodipine was performed using the C_max_ and AUC_0–72 h_. The GMR of C_max_ for amlodipine was 97.47%, and the 90% CI was 93.81%–101.26%. The GMR of AUC_0–72 h_ for amlodipine was 97.95%, and the 90% CI was 95.63%–100.31% (Table 6). The C_max_ and AUC_0–72 h_ of amlodipine were within the BE limits of 80.00%–125.00% (Figure 4D). These results indicated that the T formulation was bioequivalent to the R formulation in the postprandial state.

**TABLE 6:** Results of the equivalence determination of the T and R formulations in the postprandial trial.

Bioequivalence determination of amlodipine										

PK parameters	T		R		GMR (%)	CV_WR_ (%)	90%CI		Power (%)	Bioequivalence Criteria met?
	N	GM	N	GM						
C_max_(ng/mL)	43	3.51	84	3.6	97.47	11.83	93.81∼101.26		>99.99	YES
AUC_0–72h_(h·ng/mL)	42	114.18	84	116.58	97.95	6.52	95.63∼100.31		>99.99	YES
Bioequivalence determination of valsartan (ABE)										

PK parameters	T		R		GMR (%)	CV_WR_ (%)	90%CI		Power (%)	Bioequivalence Criteria met?
	N	GM	N	GM						
AUC_0-t_(h·ng/mL)	43	7763.17	84	7784.62	99.72	21.58	90.10∼110.37		99.82	YES
AUC_0-∞_(h·ng/mL)	42	8415.65	84	8160.51	103.13	20.19	94.81∼112.16		99.64	YES
Bioequivalence determination of valsartan (RSABE)										

PK parameters	T		R		S_WR_	CV_WR_ (%)	Point Estimate	Critbound		Bioequivalence Criteria met?
	N	GM	N	GM						
C_max_(ng/mL)	43	1273.28	84	1257.30	0.2969	30.36	102.47	−0.0453		YES

### 3.4 Safety results

Both formulations exhibited good safety in healthy Chinese participants in both fasting and postprandial states. The incidence of AEs in the fasting group was 68.89%; 71 AEs occurred in 31 participants, all of which were mild and did not require treatment. The incidence of AEs in the fasting group is summarized in Table 7.

**TABLE 7:** Summary of AEs.

Parameter	Fasting trial						Postprandial trial					
	T (N = 44)			R (N = 44)			T (N = 44)			R (N = 44)		
	*n*	*%*	*E*	*n*	*%*	*E*	*n*	*%*	*E*	*n*	*%*	*E*
sum	19	43.18	22	21	47.73	38	8	18.18	11	14	31.82	24
AE severity												
Grade 1	19	43.18	22	21	47.73	38	8	18.18	11	14	31.82	24
≥Grade 2	0	0	0	0	0	0	0	0	0	0	0	0
Correlation with drugs												
Highly Possible related	9	20.45	9	12	27.27	17	2	4.55	2	7	11.36	8
Possible related	6	13.64	8	6	13.64	9	1	2.27	1	2	4.55	3
Possible unrelated	4	9.09	5	9	20.45	12	5	11.36	8	10	22.73	14
Lymphocyte % decreased	0	0	0	1	2.27	1	0	0	0	0	0	0
ALT elevated	0	0	0	0	0	0	0	0	0	1	2.27	1
AST elevated	0	0	0	0	0	0	0	0	0	1	2.27	1
GGT elevated	1	2.27	1	0	0	0	0	0	0	1	2.27	1
U-LEU positive	1	2.27	2	1	2.27	2	1	2.27	2	0	0	0
urine erythrocyte positive	0	0	0	2	4.55	2	0	0	0	0	0	0
UOB positive	0	0	0	2	4.55	2	0	0	0	0	0	0
urinary sediment detected	0	0	0	0	0	0	1	2.27	1	0	0	0
urine nitrite detected	0	0	0	0	0	0	1	2.27	1	0	0	0
Urinary bacteria positive	0	0	0	0	0	0	1	2.27	1	0	0	0
MCHC decreased	0	0	0	1	2.27	1	0	0	0	0	0	0
Temperature elevated	1	2.27	1	4	9.09	4	0	0	0	2	4.55	2
ECG abnormality	1	2.27	1	2	4.55	2	0	0	0	2	4.55	2
Heart rate decreased	0	0	0	1	2.27	2	0	0	0	0	0	0
Serum calcium decreased	0	0	0	0	0	0	0	0	0	1	2.27	1
Serum potassium decreased	0	0	0	0	0	0	0	0	0	1	2.27	1
Serium phosphorus decreased	0	0	0	0	0	0	0	0	0	1	2.27	1
TG elevated	2	4.55	2	1	2.27	1	1	2.27	1	3	6.82	3
HGB decreased	2	4.55	2	2	4.55	2	0	0	0	1	2.27	1
Mb elevated	2	4.55	2	0	0	0	0	0	0	0	0	0
Serum creatine phosphokinase elevated	1	2.27	1	1	2.27	1	0	0	0	0	0	0
Hyperuricacidemia	0	0	0	0	0	0	2	4.55	2	2	4.55	2
Scr elevated	1	2.27	1	0	0	0	1	2.27	1	0	0	0
Blood pressure decreased	9	20.45	9	12	27.27	17	2	4.55	2	5	11.36	7
Blood pressure increased	0	0	0	1	2.27	1	0	0	0	0	0	0
GLU decreased	0	0	0	0	0	0	0	0	0	1	2.27	1
Headache	1	2.27	1	0	0	0	1	2.27	1	0	0	0
Dizziness	1	2.27	1	1	2.27	1	0	0	0	1	2.27	1
Hyperhidrosis	2	4.55	2	0	0	0	0	0	0	1	2.27	1
Acratia	1	2.27	1	1	2.27	1	0	0	0	0	0	0
Palpitation	2	4.55	2	0	0	0	0	0	0	0	0	0
Anemia	0	0	0	2	4.55	2	0	0	0	1	2.27	1
Nausea	0	0	0	0	0	0	1	2.27	1	0	0	0
Diarrhea	0	0	0	0	0	0	1	2.27	1	0	0	0

The incidence of AEs in the postprandial group was 46.67%; 42 AEs occurred in 21 subjects. All AEs were considered mild and did not require further treatment. The AEs in the postprandial group are summarized in Table 7. All AEs resolved or remained stable even after the end of the study, and results showed that the incidence and types of AEs were similar between the two formulations under fasting and postprandial conditions.

## 4 Discussion

Valsartan/amlodipine (I) is an SPC of ARB (valsartan 80 mg) and CCB (amlodipine 5 mg). Studies have shown that adherence and persistence are generally higher in patients initiating SPC anti-hypertensive medications than those taking anti-hypertensive therapy as multiple tablets in free-dose combinations (Sever et al., 2011). Additionally, valsartan/amlodipine (I) reduces major CV events and mortality in patients with hypertension by lowering blood pressure. Previous studies on valsartan/amlodipine (I) have mainly evaluated their efficacy and tolerance, and there is a lack of human studies on the PK and the influence of food on this SPC (Kizilirmak et al., 2015). This study aimed to determine the PK of valsartan and amlodipine in healthy Chinese participants to gain a better understanding of their *in vivo* characteristics. The BE between the test valsartan/amlodipine tablet (I) and the reference valsartan/amlodipine tablet (I) under fasting and postprandial states was also evaluated to provide a reference for approval in China.

Valsartan is considered a highly variable drug (HVD) because only approximately 20% is recovered as a metabolite, and cytochrome P450 isozymes are known to contribute little to its metabolism (Nakashima et al., 2005). Moreover, valsartan was revealed to be a HVD, as the CV% of the C_max_ is >30% (Wu et al., 2020). To assess the BE of HVDs, which have relatively short elimination half-lives, a replicate crossover design with three or four periods would be appropriate. A replicate crossover design can reduce the required sample size without compromising statistical power if the RSABE approach is applied in accordance with the regulatory authorities’ regulations (Tothfalusi et al., 2017; Knahl et al., 2018). Under the same conditions, a four-period replicate crossover design requires fewer participants than a three-period replicate crossover design (Endrenyi et al., 2011). Since it is considered ethical for healthy participants to participate in a short duration and low exposure to drugs, the probability of losing participants increases as the study duration becomes longer. We performed a 3-period reference replicate crossover study with 45 subjects. Statistically, the replication of administration within a subject improves the quality of the data and leads to definitive study results (Tothfalusi et al., 2017).

In this study, we used a partially replicated design with three-period by administering the R formulation twice in each sequence (TRR, RTR, and RRT). Thus, to achieve a compromise between an extended clinical phase and smaller sample size, Haidar’s method was adopted in our study (Haidar et al., 2008). Moreover, scaling to the within-subject variability of the reference formulation is a sound clinical strategy as it has already been demonstrated effective and safe in clinical practice (Dragojević-Simić et al., 2018). Therefore, it was proven to be a good selection since in a fasting state, the CV_WR_ for *C*_max_ of valsartan was >30.00% (34.68%), and the S_WR_ was 0.337 (≥0.294), while in a postprandial state, the CV_WR_% for *C*_max_ of valsartan was >30.00% (30.36%), and the S_WR_ was 0.297 (≥0.294). The CV_WR_% estimates in this study were similar to those reported in previous studies, particularly for the C_max_ for valsartan (Mansouri et al., 2017; Wu et al., 2020). The current results showed that valsartan is an HVD, and that valsartan/amlodipine (I) represents a complex problem in terms of BE assessment. A replicate crossover study can be conducted if an applicant suspects that the absorption rate and/or extent of a drug product are highly variable (Tothfalusi et al., 2009). The main purpose of our study is to support an increase in the supply of Valsartan/amlodipine (I) in China by proving the efficacy and safety of a generic alternative. For bioequivalence evaluation, we used two NMPA-specified methods (ABE and RSABE) in this study which can be used as a reference for the design and implementation of future clinical trials.

In this study, drug administration and blood collection were performed. The investigational product in our study is a SPC of amlodipine and valsartan, thus, the plasma concentrations of the two components were determined simultaneously. The absorption, distribution and elimination phases of both valsartan and amlodipine should be included in the sample collected duration, therefor, the time point design is vital in our study. Furthermore, we assessed the PK data between the fasting and postprandial trials and found that food did not influence the AUC_0–72 h_ and C_max_ values of amlodipine for either the T or R formulations. However, the main PK parameters (C_max_, AUC_0–t_, and AUC_0–∞_) of valsartan in the postprandial condition were significantly decreased compared with that in the fasting condition. We speculate that due to the differences in the structure and solubility of amlodipine and valsartan, the pharmacokinetic parameters of valsartan, which is more lipid-soluble, are easily affected by high-fat diet, but not amlodipine. The chemical name of amlodipine is 2-[(2-Aminoethoxy)methyl]-4-(2-chlorophenyl)-3-(ethoxycarbonyl)-5-(methoxycarbonyl)-6-methyl-1,4-dihydropyridine. There is an amino side chain in the molecular structure with highly water solubility. Its pKa is 8.7, which dissociates into ion form under gastric conditions and is difficult absorbed in the stomach (Zhang et al., 2017). The chemical name of valsartan is N-(1-Oxophentyl)-N-[[20-(1H-tetrazole-5-yl)[1,10-biphenyl]-4-yl] methyl]-L-valine and with highly lipid solubility. The pKa of valsartan is 4.9 and practically insoluble in water (Ardiana et al., 2015). It is difficult dissociated and easily absorbed under physiological pH conditions. Generally, food elevates the intragastric pH and decreases the rate of gastric emptying, resulting in an increased dissociation of valsartan, thereby reducing drug absorption. Moreover, physical or chemical reactions between the drugs and food may affect drug absorption after oral administration (Abuhelwa et al., 2017). However, a previous study reported that food has no effect on the absorption of valsartan and amlodipine (Sunkara et al., 2014). Inconsistent with the findings of the above studies, our study showed a significant decrease in the rate and extent of absorption of valsartan, but not of amlodipine, in the postprandial state. Various factors may contribute to this difference, including ethnicity, inter-individual differences, and metabolic enzyme genotypes. Genetic polymorphisms in metabolic enzyme genes can alter the metabolic activity toward different clinically important medications (Zhou et al., 2017). However, according to the current study, it is difficult to infer the specific influencing factors.

Despite the promising results of our trial, there were certain limitations. First, our study proved that the generic valsartan/amlodipine tablet (I) is similar to the reference valsartan/amlodipine tablet (I) in terms of PK parameters. Nevertheless, thorough studies are required to demonstrate the therapeutic BE between biosimilar and reference drugs. Another limitation was the sample size of the trial. To reduce the drug exposure of the subjects, we performed a three-period partially replicate crossover study with 45 participants. Due to the limitations of the sample size and single-dose administration, it was difficult to conduct a comprehensive assessment of the safety of the two drugs. Finally, data were collected from relatively young and healthy participants; as patients most likely to take the drug are elderly, the results should be interpreted cautiously.

## 5 Conclusion

The T formulation of valsartan/amlodipine tablet (I) was bioequivalent to the R formulation tablet under both fasting and postprandial conditions, fulfilling the requirement for BE. Food had a significant effect on the absorption of valsartan but not amlodipine. Both formulations were safe and well tolerated. This study provides a basis for a clinical trial of valsartan/amlodipine tablet (I) in the following stage and promotes the clinical application of valsartan/amlodipine tablet (I) in China.

## Data Availability

The original contributions presented in the study are included in the article/Supplementary Material, further inquiries can be directed to the corresponding authors.
